# Effect of IFN-τ on intestinal flora and metabolomics of *Escherichia coli*-mediated endometritis in mice

**DOI:** 10.3389/fmicb.2025.1687781

**Published:** 2025-12-11

**Authors:** Pan Liu, Yaofeng Zhang, Xu Chen, Lijun Tang, Wenxiu Xu, Guigui Wang, Duoqi Zhang, Junfeng Liu

**Affiliations:** 1 College of Animal Science and Technology, Tarim University, Alar, Xinjiang, China; 2 Tarim Animal Disease Diagnosis and Prevention and Control Engineering Laboratory, Xinjiang Production and Construction Corps, Aral, Xinjiang, China; 3 Sichuan Vocational and Technical College, Suining, Sichuan, China

**Keywords:** endometritis, IFN-τ, microbiome analysis, untargeted metabolomics, gut flora

## Abstract

Endometritis is a common reproductive disease in dairy cows, which can lead to low fertility or infertility and cause significant economic losses to the dairy farming industry. IFN-τ is a type I interferon that may exert significant anti-inflammatory effects in inflammatory diseases. With breakthroughs in microbial mapping sequencing and metabolomics, the role between gut flora and host metabolism and disease has been revealed from a completely new perspective. Therefore, the aim of this study was to investigate the role of IFN-τ in a mouse model of *E. coli*-induced endometritis by 16S rRNA sequencing and LC-MS untargeted metabolomics, the results showed that IFN-τ could affect the flora structure of the mouse intestine. The *E. coli*-induced endometritis in mice was found to be associated with five different metabolites and three potential metabolic pathways by LC-MS non-targeted metabolomics, which were the major players in the metabolic pathways, namely Arginine biosynthesis, Pyruvate metabolism, Arginine and proline metabolism. This may be an important metabolic pathway for IFN-τ intervention in endometritis mice. Combining the results of gut flora and metabolomics analyses suggest that changes in metabolic pathways may be influenced by gut flora. We hypothesize that IFN-τ is likely to exert its anti-inflammatory effects by regulating the levels of Oscillospira and Clostridium flora in the gut, which in turn affects the expression of five differential metabolites in uterine tissues.

## 1 Introduction

Endometritis is a common reproductive disease in female animals, leading to irreversible structural changes in the uterine wall and loss of reproductive capacity (Li et al., 2024; Smolentsev et al., 2023). Bovine endometritis poses a great challenge to the cattle breeding industry, causing significant economic losses due to reduced fertility and infertility associated with uterine inflammation, higher culling rates, and increased treatment costs (Sheldon et al., 2020; LeBlanc et al., 2002). Studies have shown that 80% to 90% of endometritis in dairy cows is caused by invasion of pathogenic bacteria (Jiang et al., 2021; Umar et al., 2021). *Escherichia coli* (*E. coli*) is a common Gram-negative bacterium that first invades endometrial cells and is considered to be the main pathogen of endometritis in domestic animals (Tao and Xuesong, 2024), which can activate the body's immune defense system through the immunostimulatory components of its cell wall, causing inflammatory responses and tissue damage in the body (Zusso et al., 2019; Zhao and Lukiw, 2018). Antibiotics are often widely used in clinical practice for the treatment of endometritis, but misuse and abuse of antibiotics often lead to antibiotic resistance (Bonet et al., 2017; Ghosh et al., 2019). Therefore, there is an urgent need for new therapeutic approaches to treat bovine endometritis.

Currently, the relationship between intestinal flora and various diseases has become a hot research topic in many scientific fields and is believed to play an important role in harmonizing the pathology and physiology of the host (Mirzaei et al., 2022). The gut microbiota consists of bacteria, fungi, protozoa, archaea, and viruses (Mirzaei et al., 2022), which encode 150 times more genes than the body's own genes. Imbalance of intestinal flora can lead to the development of many diseases, ranging from localized gastrointestinal problems to neurological, respiratory, metabolic, hepatic, and cardiovascular disorders, and a wide variety of diseases are closely related to intestinal flora imbalance (Yuzhu et al., 2024; Xiong et al., 2024; Tzu-Lung et al., 2024; Sultan et al., 2021; Pandey et al., 2022; Li et al., 2017; Cong et al., 2022). Furthermore, it has been shown that patients with endometritis have an increased abundance of harmful bacteria and a decreased abundance of beneficial bacteria in their intestinal flora (Dong et al., 2021). However, there are few reports on the relationship between intestinal flora and microbiome analysis of endometritis in dairy cows based on “intestinal flora-metabolomics”.

IFN-τ is a type I interferon secreted by trophoblast cells during ruminant embryonic development (Ma et al., 2018), which, in addition to its antiviral, antiproliferative, and immunomodulatory activity (Espin-Rivera et al., 2023), has significant low cytotoxicity and cross-species effects (Batool et al., 2025). IFN-τ has been shown to exert significant anti-inflammatory effects in inflammatory diseases (Abdelbaky et al., 2024; Liu et al., 2022; Talukder et al., 2018), However, few studies have been conducted to investigate the role of IFN-τ in *E. coli*-induced endometritis in dairy cows based on “intestinal flora-metabolomics”. Elucidating the characteristics of endometritis from microbiomic analysis and metabolomic perspectives is important for understanding its pathogenesis and potential threats.

In this study, we established a mouse endometritis model by *E. coli* induction, collected mouse uterine tissues and colon feces, and applied 16S rRNA sequencing and liquid chromatography-mass spectrometry (LC-MS) techniques to analyze the structure and composition of mouse intestinal flora and the regulation of uterine tissues' differential metabolites and related metabolic pathways among groups, and, finally, the combined 16 S rRNA and metabolomics analyses on the interaction between the differential flora and the differential metabolites. To provide a more comprehensive and detailed mechanism for elucidating the interventional role of IFN-τ in *E. coli*-mediated endometritis by modulating the organism's gut microbiota.

## 2 Materials and methods

### 2.1 Animals

36 30-day-old female Kunming-line mice weighing 18–22 g were provided by the Laboratory Center of College of Animal Science and Technology, Tarim University, then were subjected to ad libitum water and forage acclimatization feeding for 7 d under a 12-h light/dark cycle at 24±1°C, humidity of 60%.

### 2.2 Materials

Recombinant sheep interferon-tau (IFN-τ, HPLC>97%) was purchased from Beyotime Biotechnology (Shanghai, China); *Escherichia coli* (*E. coli*) ATCC 25922 (provided by Tarim Animal Disease Diagnosis and Prevention and Control Engineering Laboratory of Xinjiang Production and Construction Corps).

### 2.3 Construction of a mouse model of endometritis

Mice were randomly divided into 4 groups of 9 mice each (6 of them were sampled for testing and 3 were KEPT for reserve) to induce endometritis model: control group (CG), *Escherichia coli* group (ECO), and IFN-τ (8 μg/kg, 24 and 48 h) group (IF24 and IF48). Endometritis was modeled as follows: after 7 d of acclimatization feeding, mice were anesthetized using ketamine hydrochloride (60 mg/kg), supine held and limbs immobilized, and then *Escherichia coli* (1 × 10^6^ CFU/mL) was injected into the uterus of both sides of the mice with a 24G indwelling needle to induce endometritis. After 24 h of perfusion, the IFN-τ group was injected intraperitoneally with IFN-τ intervention for 24 and 48 h. The CG group was injected intraperitoneally with an equal amount of saline. Finally, the mice were euthanized by CO inhalation method, and uterine tissues and fecal specimens were collected and stored at −80°C.

### 2.4 16S rRNA sequencing

#### 2.4.1 Microbiome total DNA extraction

Fecal DNA was extracted using a fecal deoxyribonucleic acid (DNA) extraction kit (QIAamp Fast DNA Stool Mini Kit, purchased from China Tiangen Co., Ltd.), and the kit instructions were strictly followed. DNA was quantified using Nanodrop, and the concentration of extracted DNA was subsequently detected by 1.2% agarose gel electrophoresis, based on which DNA was diluted to 1 ng/μL in sterile water.

#### 2.4.2 PCR amplification and sequencing

The V3-V4 region of the 16S rRNA gene was amplified using universal primers (F:GTGCCAGCMGCCGCGGTAA R: GGACTACHVGGGTWTCTAAT). The PCR reaction system was 10 μL template DNA, 3 μL forward and reverse primers (10 μM), 25 μL Phusion High-Fidelity PCR Master mix with HF Buffer, and 6 μL ddH_2_O. The reaction conditions were as follows: pre-transformation at 98°C for 1 min; denaturation at 95°C for 10 s, annealing at 50°C for 30 s, and extension at 72°C for 30 s for a total of 25 cycles; and finally, holding at 72°C for 5 min and storage at 4°C.PCR products were detected by 1.2% agarose gel electrophoresis.After creation of sequencing libraries using Illumina's TruSeq Nano DNA LT Library Prep Kit, the libraries were quantified by Quant-iT PicoGreen dsDNA Assay Kit on a Promega QuantiFluor Fluorescence Quantification System and purified using AMPure XP beads (Beckman Coulter, Indianapolis, Indiana) for purification.Sequencing was performed using the NovaSeq 6000 SP Reagent Kit (500 cycles).

### 2.5 LC-MS untargeted metabolomics

#### 2.5.1 Uterine tissue sample extraction

1) Remove the uterine tissue sample to be assayed from the −80 °C refrigerator and thaw it on ice to ensure accurate weighing of the tissue sample;
2) Blood was aspirated from the surface of the samples using filter paper and the desired sample of uterine tissue was cut off using a scalpel, each sample needed to be weighed 30±2 mg and the weight of each sample was recorded;
3) Add 500 ul, methanol acetonitrile mixture (1:1/V) to the EP tube of the sample that has been weighed and add a steel bead, put it into the grinder and grind it 6 times until it is homogeneous;
4) Samples were placed in liquid nitrogen for 1 min, thawed at room temperature, sonicated for 10 min and repeated twice;
5) Centrifuge at 4 °C, 17,000 g/min, for 15 min, remove the supernatant and inject into the appropriate EP tube;
6) 35 °C, vacuum centrifuged and drained;
7) The sample was added to 100 ul of 50% methanol water and vortexed for 60 s;
8) Ultrasonic treatment for 10 min;
9) Centrifuge the sample for 10 min at 20 °C again using a centrifuge at 17,000 g/min;
10) After centrifugation, 200 uL of the supernatant was injected into the liner tube of the corresponding injection bottle and analyzed on the machine.

#### 2.5.2 Chromatographic and mass spectrometric conditions

The AB 5600 Triple TOF mass spectrometer is capable of primary and secondary mass spectrometry data acquisition based on IDA functionality under the control software (Analyst TF 1.7, AB Sciex). In each data acquisition cycle, the molecular ions with the strongest intensity and greater than 100 were selected for acquisition of the corresponding secondary mass spectrometry data. Primary acquisition range 50–1,200, bombardment energy: 30 eV, 15 secondary spectra per cycle. Among them, the liquid phase conditions include the following:

The column was a Waters ACQUITY UPLC HSS T3 C18 1.8 μm, 2.1 mm^*^100 mm; The mobile phase was divided into two, phase A was ultrapure water (0.1% formic acid) and phase B was acetonitrile (0.1% formic acid); The elution gradient was performed as follows: water/acetonitrile (95:5 V/V) at the beginning, gradually changing to 10:90 V/V after 10 min for 1 min, and then reverting to a water/acetonitrile ratio of 95:5 V/V again at 11.1 min, for a total detection time of 14 min; The liquid-phase flow rate was set at 0.4 mL/min, the column temperature was set at 40°C, and the injection volume was 4 μL.

The mass spectrometry conditions were:

1) The temperature of the electrospray ionization (ESI) source was set to 500 °C;
2) In positive and negative ion mode, the mass spectral voltages were 5,500 and −4,500 V, respectively;
3) Sample molecules are transported to the mass spectrometer under ion source gas I (GS I) at 60 psi, gas II (GS II) at 60 psi, and curtain gas (CUR) at 35 psi. gas (CUR) at 35 psi, the sample molecules form an ion gas cloud and are transported to the mass spectrometer;
4) The collision-activated dissociation (CAD) parameter was set to high;
5) Finally, in a triple quadrupole (Qtrap), each ion pair was scanned and detected according to the optimized declustering voltage (declustering potential, DP) and collision energy (collision energy, CE). potential (DP) and collision energy (CE) for scanning detection.

### 2.6 Statistical analysis

The valid sequences of all samples were clustered at 97% similarity using Uparse software to obtain OTUs. On this basis, the interspecific relationship indices within each taxon were calculated separately, and their distribution was used to determine whether they belonged to the same biological taxon. The OTU sequences were analyzed for species annotation using the Mothur method with the SSUrRNA database from SILVA138, and this information was used to count the community composition of each sample. The QIIME software was used to generate species abundance tables at different taxonomic levels, which were then plotted as community structure maps at each taxonomic level of the samples using R language tools. Sequences with the highest abundance of features at the genus taxonomic level were selected as representative sequences using the QIIME software, multiple sequence comparison was performed and phylogenetic trees were constructed and plotted graphically by the Python language tools. Using QIIME2 software, the Alpha and Beta diversity indices of the samples were evaluated, and the Wilcoxon Rank-Sum Test was used to analyze the differences between groups of diversity indices, and combined with the multiple testing method to screen for differential bacteria, and the significance level of *P* < 0.05 was considered to be a significant difference.

The mass spectrometry data were processed using the software Analyst 1.6.3 and the metabolites of the samples were analyzed qualitatively and quantitatively by mass spectrometry based on the local metabolic database. PCA was performed with the built-in statistical prcomp function of the R software (www.r-project.org/), setting the prcomp function parameter scale=True to indicate unit variance scaling (UV) normalization of the data. Metabolites with significant differences between groups were screened based on FC (fold change) ≥2 or ≤ 0.5 and VIP (Variable importance of projection) >1. Identified metabolites were labeled using the KEGG Compound Database (http://www.kegg.jp/kegg/compound/), and then the screened metabolites were mapped to the KEGG Pathway Database (http://www.kegg.jp/kegg/pathway.html). Finally, metabolite pathways with significant differences were mapped to metabolite enrichment analyses and significance was determined by *p*-values from hypergeometric tests.

The experimental data were statistically analyzed using SPSS 20.0 software. Mean ± standard deviation ($\bar{x}$ ± S) was used to describe the measurement data. The *t*-test was used to compare the data between the two groups, and one-way ANOVA was used to compare the differences between the data of multiple groups. *P* < 0.05 indicated that the differences were statistically significant, and *P* < 0.01 indicated that the differences were significant.

## 3 Results

### 3.1 Intestinal flora results

#### 3.1.1 Number and distribution of OTUs

**Figure 1:**
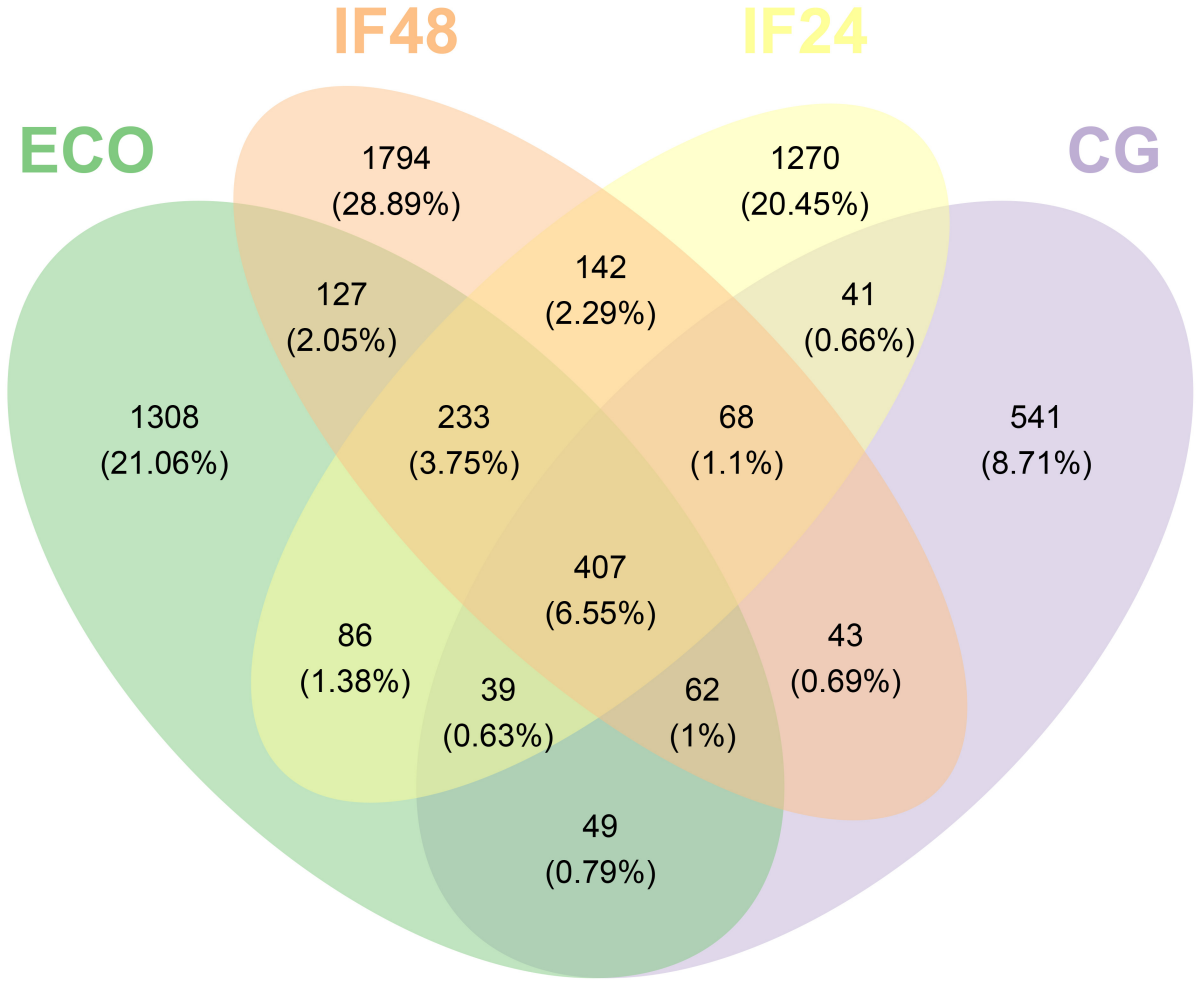
OTU distribution diagram between five groups.

The uniform grouping of sequences obtained by sequencing with 97% similarity is called an operational taxonomic unit (OTU) and is important for analyzing species diversity. Analyzing the number of shared and unique OTUs between different groups helps in subsequent Alpha diversity and Beta diversity analyses. As shown in Figure 1, the number of OTUs shared by the four groups was 407, and the number of OTUs specific to the CG, ECO, IF24, and IF48 groups were 541, 1,308, 1,270, and 1,794, accounting for 8.71, 21.06, 20.45, and 28.89% of the total OTUs, respectively. The results showed that the percentage of unique OTUs was higher in the four subgroups, the community composition of unique flora in different subgroups had a large difference, and the IFN-τ intervention was able to affect the OTU composition of the intestinal flora in endometritis mice.

#### 3.1.2 Alpha diversity analysis

**Figure 2:**
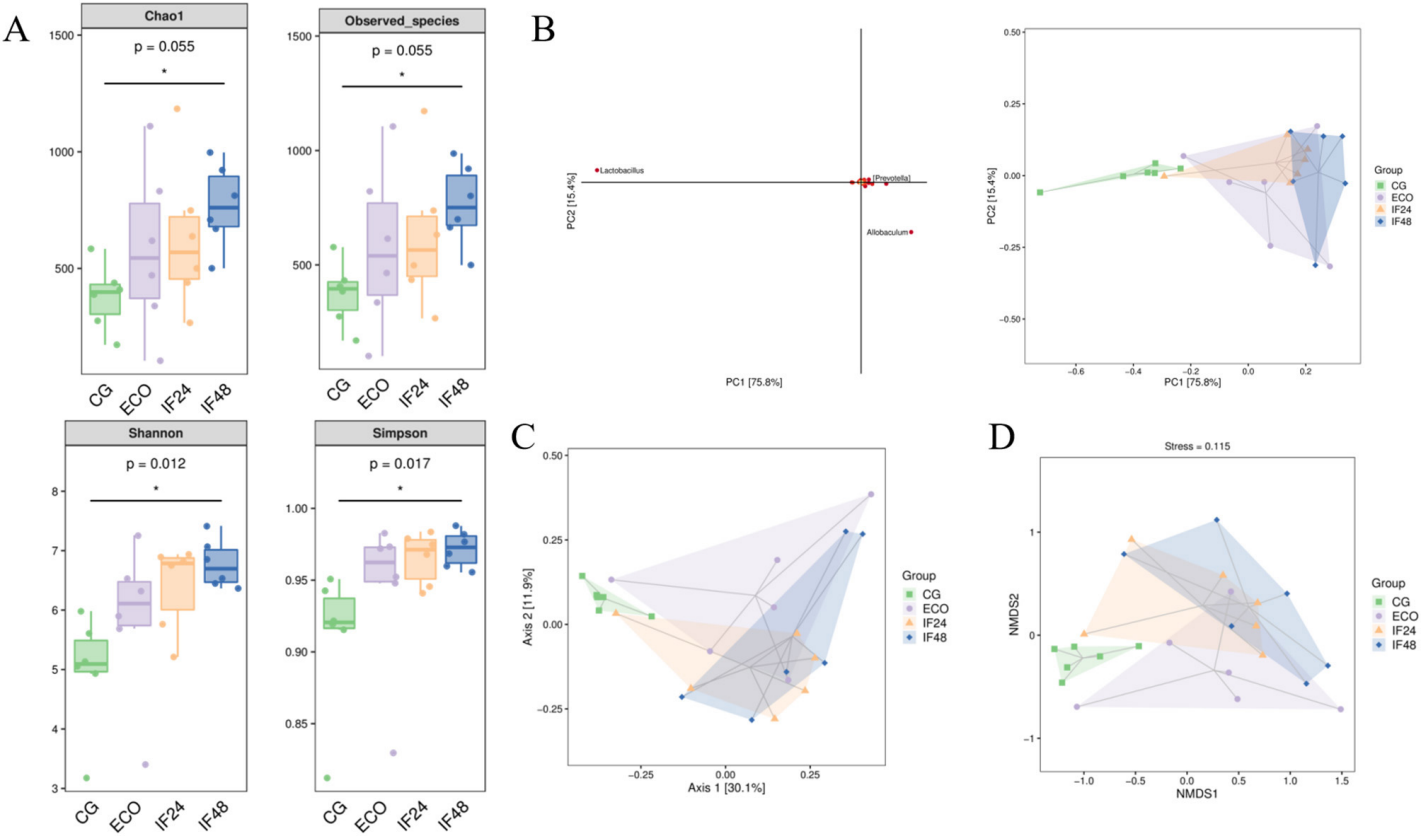
Alpha and Beta diversity analy sis. **(A)** Alpha diversity index. **(B)** Principal component analysis. **(C)** PCoA analysis based on Bray-Curtis distance. **(D)** NMDS analysis based on Bray-Curtis distance.

For Alpha diversity analysis, Chao1 and Observed species indices were used to analyze bacterial community abundance and Shannon and Simpson indices to analyze community diversity. As shown in Figure 2A, the CG, ECO, IF24, and IF48 groups exhibited statistically significant differences in the Chao1 index (*P* = 0.055), observed species index (*P* = 0.055), Shannon index (*P* = 0.012), and Simpson index (*P* = 0.017). The results showed that similar richness, different diversity.

#### 3.1.3 Beta diversity analysis

Analyzed by PCA (Figure 2B, PC 1 and 2 accounted for 75.8% and 15.4% of the total change, respectively), PCoA principal coordinates (Figure 2C, PCoA 1 and 2 accounted for 30.1 and 11.9% of the total change, respectively) and NMDS (Figure 2D) showed that the separation between the CG group and the ECO group was obvious, indicating that the intestinal flora of the mice in the ECO group was significantly altered; there was a certain distance between the IF24 group and the IF48 group and the ECO group, suggesting that IFN-τ intervened to change the composition of the intestinal flora of the mice, but this change was not consistent with that of the mice in the CG group.

#### 3.1.4 Analysis of the species composition of intestinal microorganisms

**Figure 3:**
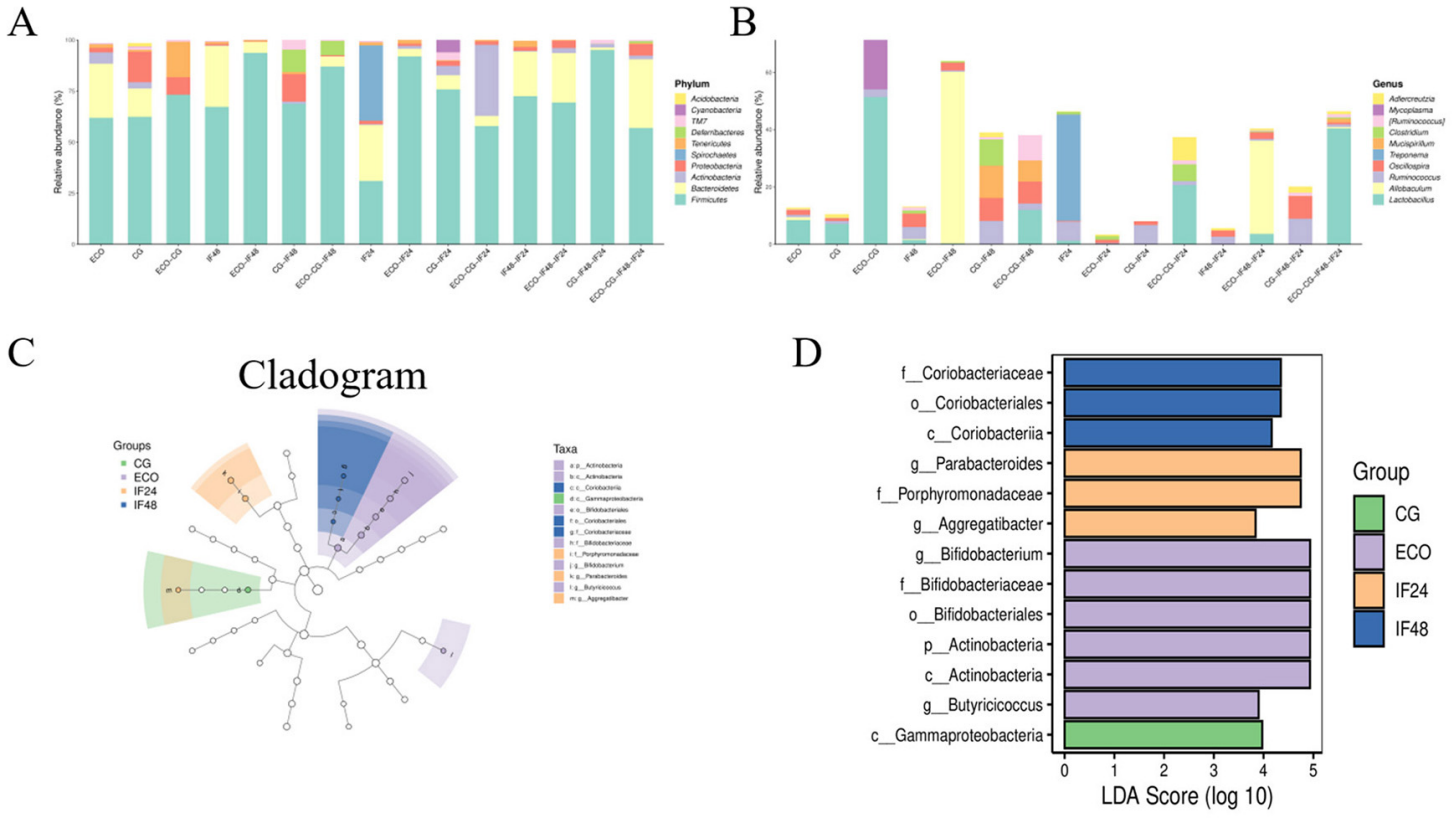
Analysis of the species composition of gut microorganisms and analysis of species differences. **(A)** Species composition at the phylum level. **(B)** Species composition at genus level. **(C)** Lefse branching diagram of gut microbiota. **(D)** LDA value distribution diagram.

Comparison of microbial reference databases with representative sequences from OTUs allows for the acquisition of taxonomic information on species corresponding to OTUs, which in turn counts the community composition of the samples at the phylum and genus taxonomic level. As shown in Figure 3A, there was a significant difference in gut flora between the groups. The top 10 phyla and genera with the highest average ASV/OTU frequencies in the sample were taken from each group for presentation, and the results showed that *Firmicutes, Bacteroidetes*, and *Proteobacteria* were the top abundance groups in the four groups. The abundance of *Proteobacteria* was higher in the CG group compared to the ECO group. Compared with the ECO group, *Bacteroidetes*, abundance was significantly increased and *Proteobacteria* abundance was decreased after 24 h of IFN-τ addition. This suggests that also in the case of *E. coli* stimulation, IFN-τ interference can reduce endotoxin damage to the organism by increasing the abundance of beneficial flora decreasing the abundance of harmful flora. According to the pharmacokinetic study of recombinant sheep interferon-tau (roIFN-τ) by Zhao et al. (2019), this molecule exhibits a short duration of action in lambs, with its activity failing to persist for 48 h. However, our experimental data indicate that IFN-τ treatment significantly increased the abundance of Proteobacteria in the gut microbiota after 48 h. This seemingly contradictory phenomenon may suggest that although circulating IFN-τ concentrations have declined, it may continue to influence the intestinal microenvironment through activated immune signaling pathways or induced host defense peptides, thereby selectively promoting the proliferation of Proteobacteria. Figure 3B compares the species composition of the intestinal flora and its changes in mice in the CG, ECO, IF24 and IF48 groups at the “genus” level of classification. The results showed that *Lactobacillus* was the predominant bacterium in each group, and *Lactobacillus* was not only enriched in the ECO group compared to the CG group, but also enriched in the ECO group compared to the IF24 and IF48 groups. Compared to the ECO group, *Oscillospira* was elevated after 24 h of IFN-τ addition, and *Allobaculum* became the dominant bacterial group in the IF24 group after 24 h of IFN-τ addition compared to the CG group. Compared to the ECO group, *Clostridium* and *Oscillospira* were increased 48 h after the addition of IFN-τ. *Allobaculum* became a dominant bacterial group in the IF48 group compared to the CG group.

#### 3.1.5 Analysis of species differences

LEfSe analysis was used in this study to identify the biomarkers that matched based on the screening criteria set for the biomarkers (LDA score > 2) and presented graphically; the significance of the difference between two-by-two subgroups at each level of classification was compared. The results showed that a total of 13 statistically different biomarkers were identified in the four groups (Figures 3C, D). The representative bacterial groups varied between groups, with higher levels of c_*Gammaproteobacteria* found in the CG group; higher levels of g_*Bifidobacterium*, g_*Butyricoccus* in the ECO group; and g_*Parabacteroides* dominating in the IF24 group; The IF48 group was dominated by f_*Coriobacteriaceae*.

#### 3.1.6 Prediction of intestinal flora function

**Figure 4:**
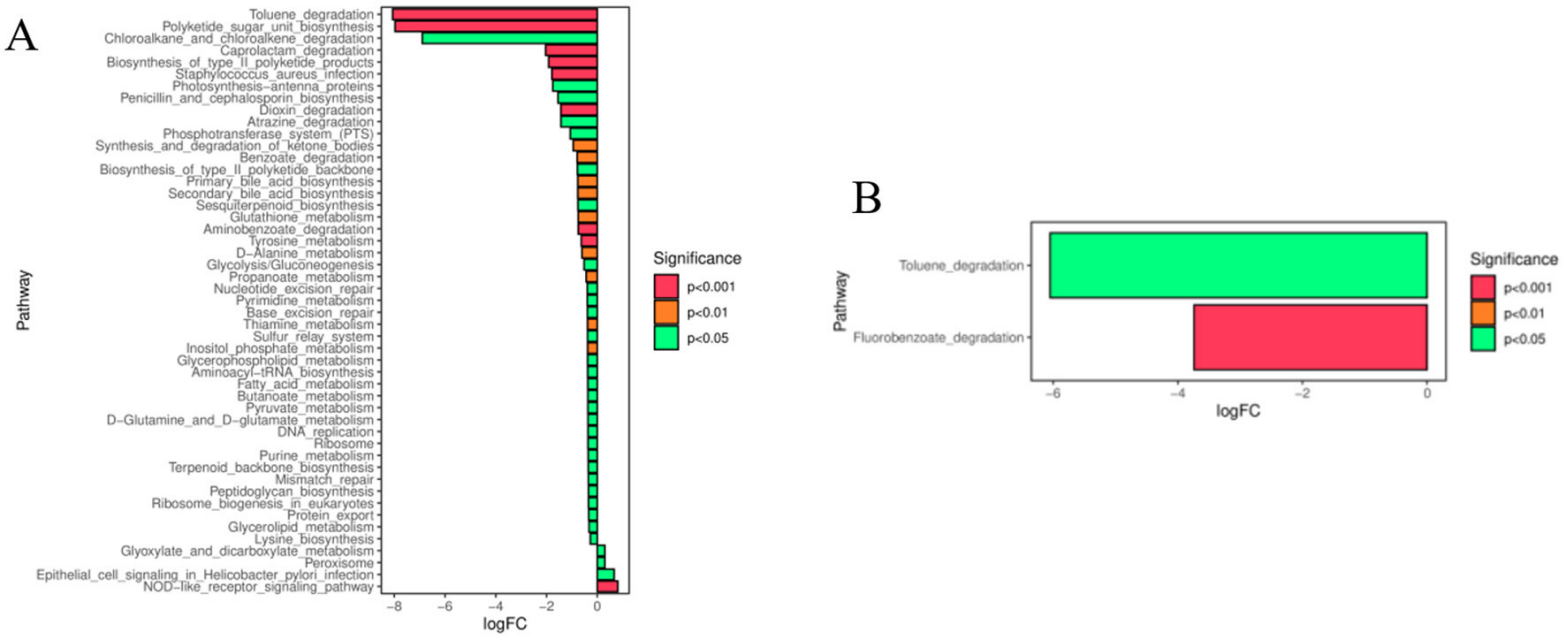
Prediction of functional pathways of gut microbiota differences between CG and IF48 groups **(A)** and ECO and IF48 groups **(B)**.

To investigate the functional changes of intestinal flora in endometritis mice after IFN-τ intervention, flora function prediction was performed based on the KEGG database, and the pathways that were altered in both the CG and IF48 groups, and the ECO and IF48 groups, could be regarded as differential pathways. As shown in Figure 4A, the CG group and the IF48 group existed Toluene degradation, Polyketide sugar unit biosynthesis, Chloroalkane and chloroalkene degradation, Caprolactam degradation, Propanoate metabolism, Glyoxylate and dicarboxylate metabolism, Aminobenzoate degradation, Glycolysis/Gluconeogenesis, Peptidoglycan biosynthesis, NOD-like receptor signaling pathway, *Staphylococcus aureus* infection and other metabolic pathway differences. As shown in Figure 4B, there were differences in the metabolic pathways of Toluene degradation, Fluorobenzoate degradation between the ECO group and the IF48 group.

### 3.2 Metabolomics results

#### 3.2.1 Experimental quality control

**Figure 5:**
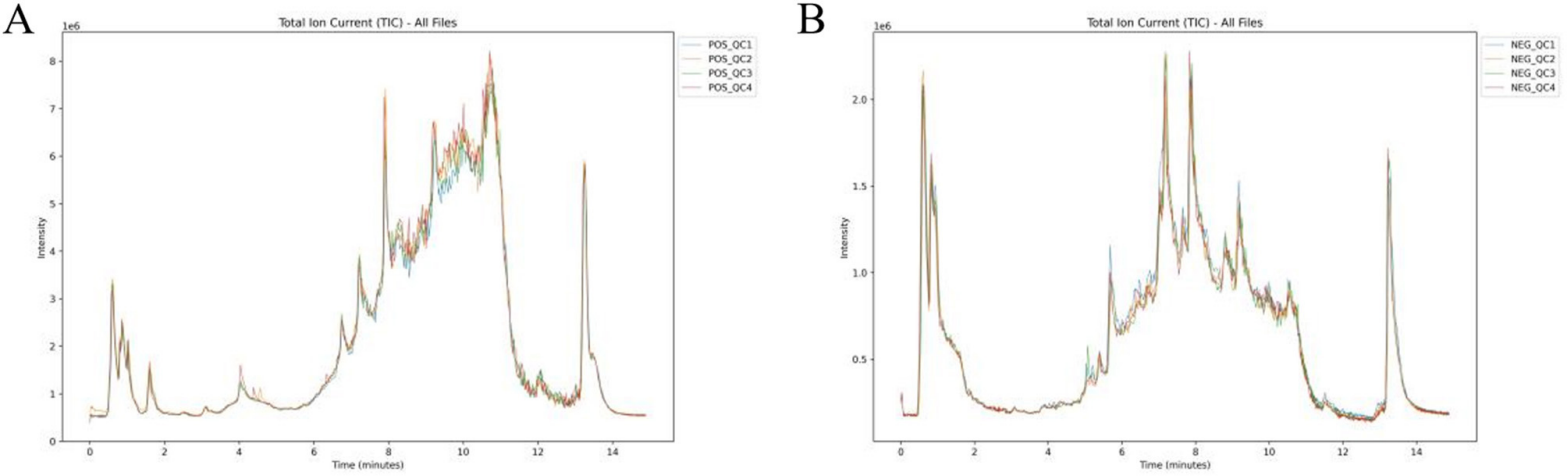
TIC of QC samples. **(A)** Positive Total Ion Chart TIC. **(B)** Negative Ion Total Ion Chart TIC.

QC analysis result display: Overlapping display analysis of different Quality Control (QC) samples (TIC) by Total Ion Chromatogram (TIC), thus ensuring data stability and reliability. As shown in Figure 5, the overlap of the curves is high in both positive and negative ion modes, the retention time and peak intensity are consistent, and the instrument has high stability.

#### 3.2.2 Subgroup principal component analysis

**Figure 6:**
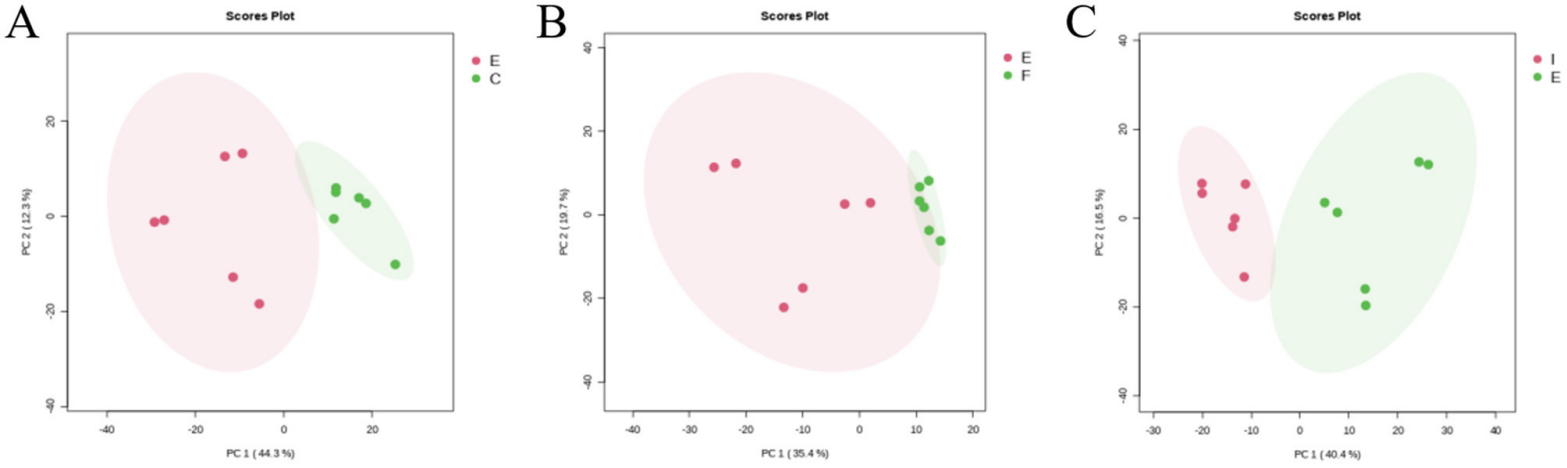
PCA analysis of ECO group and CG, IF24, IF48 groups. **(A)** ECO group and CG group. **(B)** ECO group and IF24 group. **(C)** ECO group and IF48 group.

Subgroup PCA analyses were performed on the ECO group (E) vs. the CG group (C), the IF24 group (F), and the IF48 group (I) to reflect the degree of variability between groups and metabolic differences between groups. As shown in Figure 6, in the positive and negative dual ion mode, the PCA plot showed that among the IFN-τ-treated groups, the ECO group was significantly differentiated from the IF48 group, and the ECO group was not significantly differentiated from the IF24 group, indicating that the endogenous metabolites were altered by IFN-τ treatment for 48 h. The endogenous metabolites of the IFN-τ-treated groups were also differentiated from those of the IF24 group.

#### 3.2.3 Orthogonal partial least squares discriminant analysis

**Figure 7:**
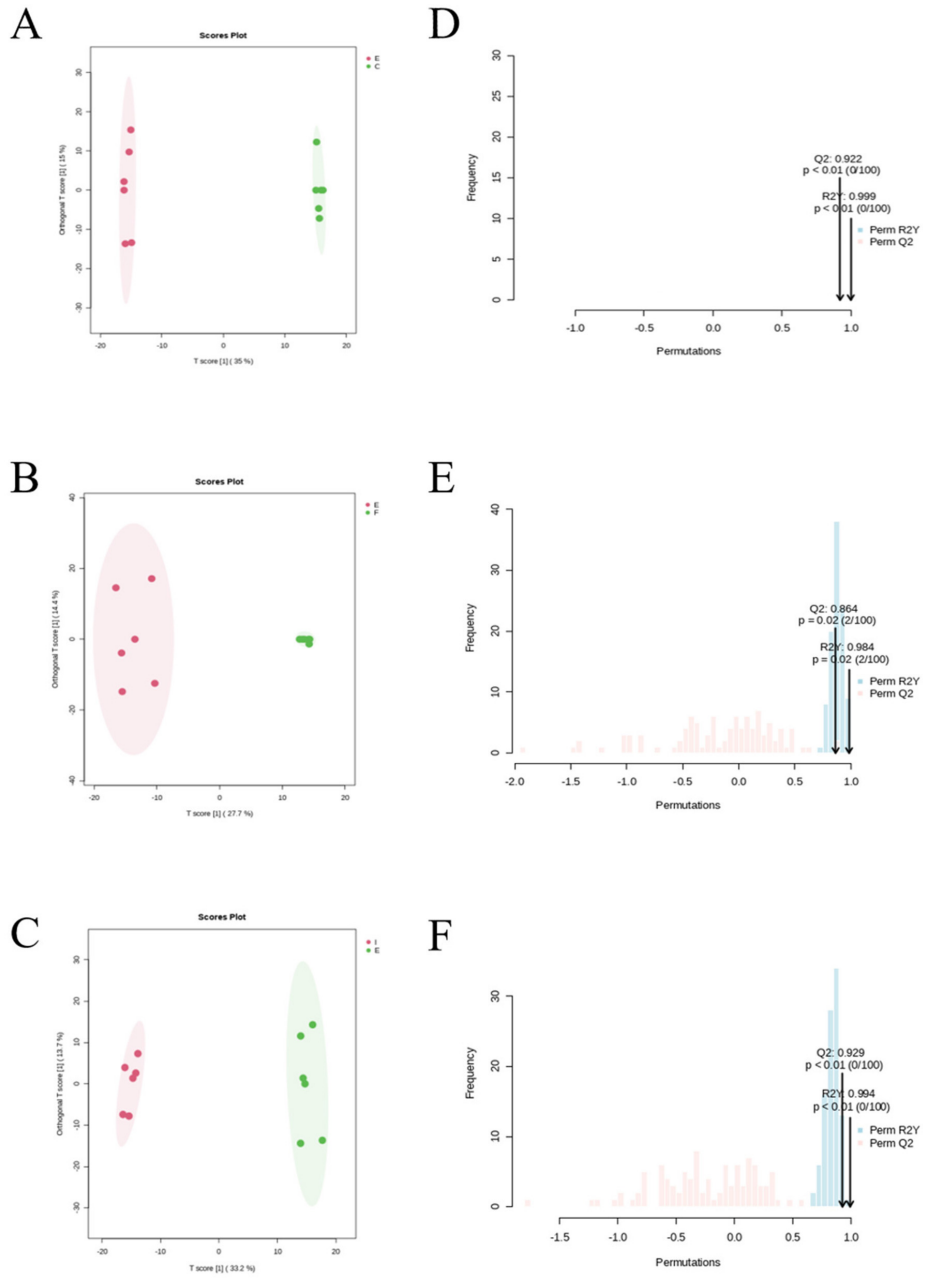
OPLS-DA score plots and permutations of differential. **(A)** OPLS-DA score plot between ECO and CG. **(B)** OPLS-DA score plot between IF24 and ECO. **(C)** OPLS-DA score plot between IF48 and ECO. **(D)** OPLS-DA model validation plot between ECO and CG. **(E)** OPLS-DA model validation plot between IF24 and ECO. **(F)** OPLS-DA model validation plot between IF48 and ECO (P <0.05).

Metabolic outcomes were analyzed between groups using the OPLS-DA model for the CG group (C), the IF24 group (F), the IF48 group (I) & the ECO group (E), respectively. The model was first evaluated and the results of the evaluation of CG, ECO, IF24 and IF48 groups are shown in Figures 7D–F. The results showed a significant separation between CG and ECO groups, which indicated that the metabolites of endometritis mice were significantly changed. In addition, the OPLS-DA model parameters showed R2Y = 0.999 and Q2 = 0.922 > 0.5, which indicates that the classification model has good predictive ability and goodness of fit, and the data description is reliable. There was also a significant separation between the ECO group and the IF24 and IF48 groups, with OPLS-DA model parameters of R2Y = 0.984, Q2 = 0.864 > 0.5, and R2Y = 0.994, Q2 = 0.929 > 0.5, respectively, which suggests that the classification model has good predictive power and goodness of fit, and that the data description is reliable. In the PC1 dimension, the groups of samples were completely separated. In summary, the results of the statistical analysis of the samples using OPLS-DA showed that the metabolites of endometritis mice changed significantly, and the OPLS-DA model was able to classify the samples effectively, and the Q^2^ were all greater than 0.5 indicating that the model had a better prediction of the differential metabolites in each comparison group.

The OPLS-DA score graphs of CG, IF24, IF48 and ECO groups are shown in Figures 7A–C, respectively. The results showed that the samples within each group were within the confidence interval, the distribution was more concentrated, there was similarity in the samples, and the model could be better applied to visualize and analyze the differences in metabolite expression between groups.

#### 3.2.4 Uterine differential metabolite screening

**Figure 8:**
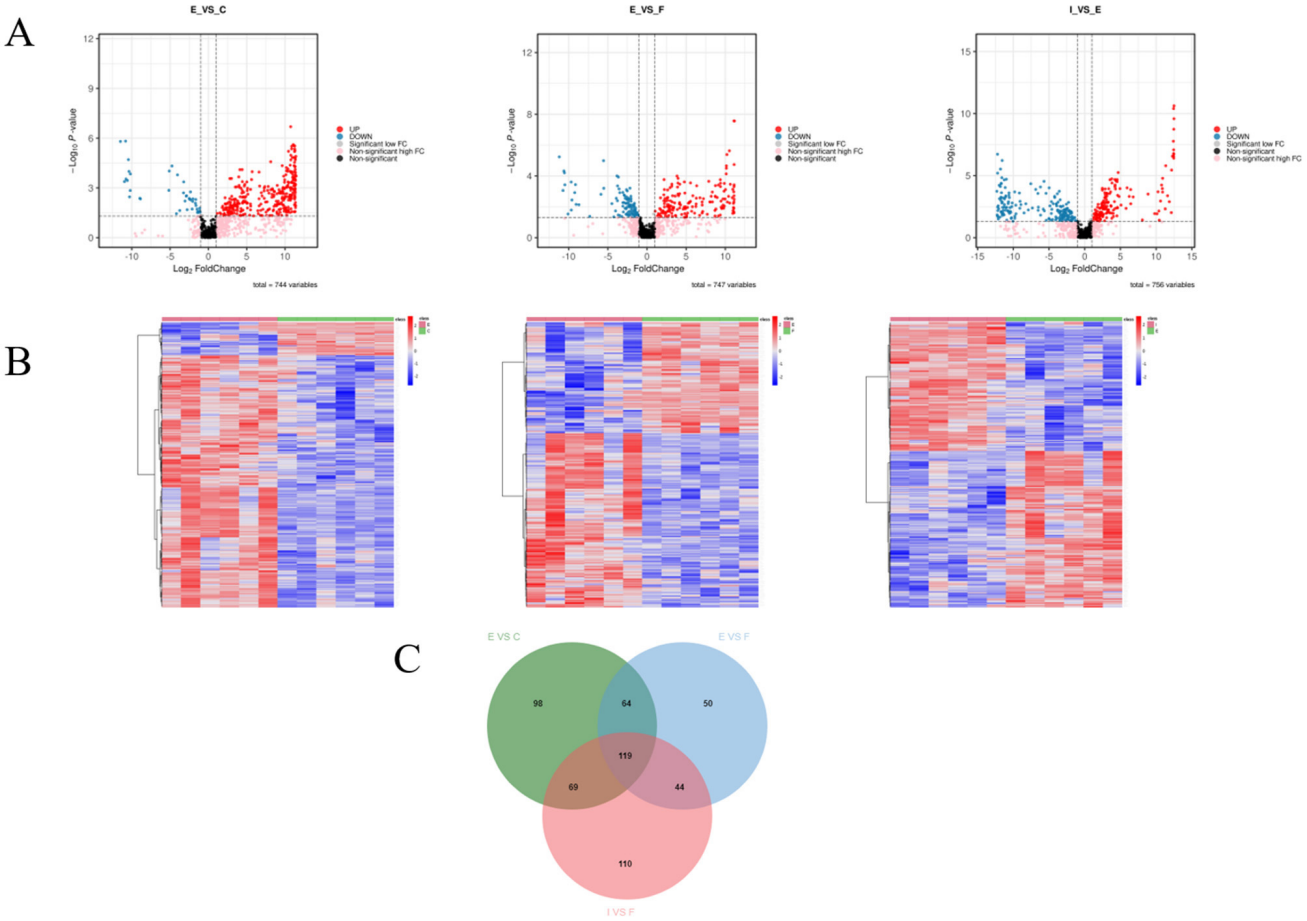
Differential metabolite screening and analysis. **(A)** Volcanic maps showed the expression levels of different differential metabolites in CG and ECOgroups **(left)** ECO and IF24 groups **(middle)** and IF48 groups **(right)**. Blue dots represent down-regulated differentially expressed metabolites, red dots represent up-regulated differentially expressed metabolites, and black dots represent detected but not significantly different metabolites. **(B)** Visual heat map. The different metabolites of CG group and ECO group **(left)**, ECO group and IF24 group **(middle)**, ECO group and IF48 group were normalized **(right)**. Horizontal is grouping information, vertical is differential metabolite information, cluster tree on the left is differential metabolite cluster tree, Scale is the expression quantity obtained after standardized processing (the redder the color, the higher the expression quantity). **(C)** Venn diagram showed the common and unique differences of metabolites between CG and ECO, ECO and IF24, ECO and IF48.

We first screened the intergroup difference metabolites between each group and the ECO group separately, using log2FC ≥ 1, VIP value ≥ 1, and *P* < 0.05 as criteria, and the results are shown in Figure 8. The results showed that 41 metabolites were significantly up-regulated and 309 metabolites were significantly down-regulated in the ECO group of mice compared to the CG group (Figure 8A left); Compared with the ECO group, 108 metabolites were significantly up-regulated and 169 metabolites were significantly down-regulated in the IF24 group of mice (Figure 8A in); Compared with the ECO group, 184 metabolites were significantly up-regulated and 158 metabolites were significantly down-regulated in the IF48 group of mice (Figure 8A right). 119 metabolites were all found in the CG group vs. the ECO group, the ECO group vs. the IF24 group, and the ECO group vs. the IF48 group (Figure 8C). Suggests that IFN-τ significantly regulates the expression of metabolites in endometritis mice.

Then, the metabolites in each group were analyzed qualitatively and quantitatively, and the top 25 intergroup differential metabolites between each group and the ECO group were obtained by using log2FC ≥ 1 as the screening criterion, as shown in Tables 1, 2, respectively, in which 6 metabolites were found in both the CG group and the ECO group, as well as the ECO group and the IF24 group. 5 metabolites were found in both the CG and ECO groups and the ECO and IF48 groups. As shown in Table 2, compared with the CG group, the ECO group had elevated levels of the differential metabolites of Propane-1,3-diyl bis(4-aminobenzoate), Nitrendipine, and 2-Naphthalene sulfonic acid; and reduced levels of 2-Deoxytidine diphosphate (dCDP), Glutathione differential metabolite content was decreased; however, the levels of the above 5 metabolites in the IF48 group compared to the ECO group were in contrast to the ECO vs. CG group. 1 metabolites were all found in the CG group vs. ECO group, the ECO group vs. IF24 group, and the ECO group vs. IF48 group. As shown in Tables 1, 2, Propane-1,3-diyl bis(4-aminobenzoate) content was elevated in CG group vs. ECO group and ECO group vs. IF24 group, and Propane-1,3-diyl bis(4-aminobenzoate) content was reduced in ECO group vs. IF48 group. Apparently, 5 metabolites, Propane-1,3-diyl bis(4-aminobenzoate), Nitrendipine, 2-Naphthalene sulfonic acid, 2-Deoxytidine diphosphate (dCDP), and Glutathione, were the important metabolites for IFN-τ intervention in endometritis mice.

**Table 1:** Differential metabolites in CG vs. ECO and ECO vs. IF24 groups.

**Metabolites**	**Groups**	**VIP**	**log2(FC)**	**Trends**
Vanillin-4-sulfate	ECO vs. CG	1.58	11.28	↑
	IF24 vs. ECO	1.65	3.95	↑
Sericetin	ECO vs. CG	1.59	10.45	↑
	IF24 vs. ECO	1.81	10.21	↑
Propane-1,3-diyl bis (4-aminobenzoate)	ECO vs. CG	1.56	11.34	↑
	IF24 vs. ECO	2.18	1.61	↑
Isoxanthohumol	ECO vs. CG	1.56	10.76	↑
	IF24 vs. ECO	1.82	10.52	↑
Difucol Hexamethyl Ether	ECO vs. CG	1.55	8.13	↑
	IF24 vs. ECO	1.73	7.89	↑
Docosahexaenoyl Glycune	ECO vs. CG	1.53	9.63	↑
	IF24 vs. ECO	1.70	9.39	↑

**Table 2:** Differential metabolites in CG vs. ECO and ECO vs. IF48 groups.

**Metabolites**	**Groups**	**VIP**	**log2(FC)**	**Trends**
Propane-1,3-diyl bis (4-aminobenzoate)	ECO vs. CG	1.56	11.34	↑
	IF48 vs. ECO	6.73	−12.21	↓
Nitrendipine	ECO vs. CG	1.57	11.33	↑
	IF48 vs. ECO	1.63	−12.20	↓
2-Naphthalene sulfonic acid	ECO vs. CG	1.64	10.74	↑
	IF48 vs. ECO	1.60	−11.61	↓
2-Deoxytidine diphosphate (dCDP)	ECO vs. CG	1.62	−10.81	↓
	IF48 vs. ECO	1.67	11.34	↑
Glutathione	ECO vs. CG	1.55	−10.44	↓
	IF48 vs. ECO	1.60	10.91	↑

#### 3.2.5 Analysis of differential metabolite pathways

**Figure 9:**
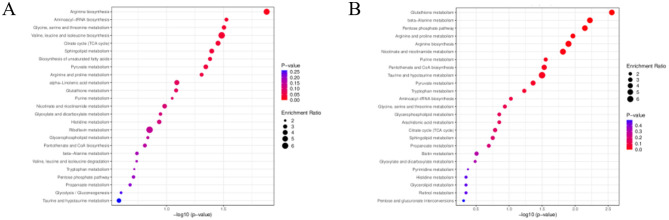
KEGG enrichment analysis of differential metabolites. KEGG enrichment of differential metabolites in CG group versus ECO group **(left)** in ECO group versus IF48 group **(right)**. Enrichment Ratio is the ratio of the number of differentially expressed metabolites in the corresponding pathway to the total number of metabolites detected in the pathway; the larger the ratio, the higher the enrichment degree of the pathway. The size of the dots reflects the number of differentially significant metabolites enriched in the corresponding pathway; the color represents the magnitude of the P-value value, with redder indicating more significant enrichment.

KEGG metabolic pathway enrichment analysis of differential metabolites in endometritis mice was performed, and the metabolic pathway results obtained were sorted according to the *P* value from smallest to largest, and the top 25 results are shown in Figure 9, after which the differential pathways were analyzed in each group using a *P* value of less than 0.05 as the screening criterion. Differential metabolites between the CG and ECO groups were significantly enriched in 9 metabolic pathways, including Arginine biosynthesis, Aminoacyl-tRNA biosynthesis, Glycine, serine and threonine metabolism, Valine, leucine and isoleucine biosynthesis, Citrate cycle (TCA cycle), Sphingolipid metabolism, Biosynthesis of unsaturated fatty acids, Pyruvate metababolism and isoleucine biosynthesis, Citrate cycle (TCA cycle), Sphingolipid metabolism, Biosynthesis of unsaturated fatty acids, Pyruvate metabolism, Arginine and proline metabolism. Differential metabolites between the ECO and IF48 groups were significantly enriched mainly in 10 metabolic pathways, includingGlutathione metabolism, beta-Alanine metabolism, Pentose phosphate pathway, Arginine and proline metabolism, Arginine biosynthesis, Nicotinate and nicotinamide metabolism, Purine metabolism, Pantothenate and CoA biosynthesis, Taurine and hypotaurine metabolism, Pyruvate metabolism. There were three main common metabolic pathways enriched between the CG group and the ECO group and between the ECO group and the IF48 group, which were Arginine biosynthesis, Pyruvate metabolism, and Arginine and proline metabolism.This may be an important IFN-τ intervention in endometritis mice metabolic pathway.

## 4 Discussion and conclusions

Endometritis is an inflammation of the endometrial layer of the uterus that usually occurs at 21 d postpartum and severely affects the productivity and reproductive performance of affected cows, resulting in large economic losses in dairy farming (Wagener et al., 2017). Antibiotics play a vital role in livestock production as a powerful “weapon” against infectious diseases (Pan et al., 2024). However, the prolonged and massive use of antibiotics inevitably leads to problems such as antibiotic accumulation and bacterial resistance (Du et al., 2022; Chiesa et al., 2019). Therefore, it is urgent to find other drugs to replace antibiotics. IFN-τ has been shown to have excellent anti-inflammatory effects (Abdelbaky et al., 2024; Liu et al., 2022; Talukder et al., 2018). Previous studies by the group have demonstrated that IFN-τ can inhibit the inflammatory response response and apoptosis of bEECs and contain the inflammatory injury of endometrial tissues in dairy cows by up-regulating the post-transcriptional regulation of miR-505 to mediate the HMGB1/NF-κB signaling pathway (Junfeng, 2021). However, few articles have examined how the gut flora and uterine tissue metabolites of animal organisms with endometritis interact with IFN-τ intervention.

In the mouse endometritis model we successfully established in this study, 16S rRNA sequencing revealed structural changes in the gut microbiota of endometritic mice. In addition, we found that IFN-τ could affect the OTU composition of the intestinal flora of mice; Alpha diversity indicated that IFN-τ altered the intestinal flora diversity; Beta diversity indicated that the intestinal flora of endometritis mice changed significantly. Analysis of the intestinal flora composition of mice at the genus level showed that IFN-τ could alter the intestinal flora composition, and its mechanism of action was mainly to restore the microecological structure of the intestinal microbiota by regulating the growth of the *Oscillospira* and *Clostridium* flora and then restoring the microecological structure of the intestinal microbiota. Research findings by Zhao et al. (2022) indicate that microbe-mediated AhR activation plays a crucial role in the pathogenesis of endometritis, offering potential strategies for intervening in infectious diseases and reproductive health by modulating the gut microbiota and metabolism. Research findings by Xue et al. (2023) indicate that IFN-τ alleviates damage caused by Escherichia coli-induced endometritis in mice and improves the endometrial tissue barrier. Its mechanism of action may involve reducing the abundance of Enterobacteriaceae in the gut microbiota and influencing the expression levels of key metabolites in uterine tissue, thereby exerting anti-inflammatory effects. It has been shown that Treg cells are negative regulators of inflammation and that microbial species of *Clostridium* clusters XIVa, IV and XVIII can alter regulatory T cells (Treg) and T helper cells 17 differentiation and thus maintain intestinal immune homeostasis (Atarashi et al., 2011, 2013; Senchukova, 2023). Functional dietary fiber promoted the enrichment of *butyric acid-producing bacteria-Oscillospira spp*. as well as the production of butyric acid, which reduced intestinal permeability and plasma endotoxin levels in sows during the periparturient period (Chuanshang, 2019). In addition, dietary fiber may improve the insulin resistance status caused by high-fat and high-sugar diets by modulating the relative abundance of intestinal *Oscillospira* in rats in the high-sugar and high-fat diet group, which in turn alters the LPS content in the organism (Yanyang et al., 2022). LC-MS non-targeted metabolomics study showed that the levels of Propane-1,3-diyl bis(4-aminobenzoate), Nitrendipine, 2-Naphthalene sulfonic acid, 2-Deoxytidine diphosphate (dCDP), and Glutathione were significantly increased, and IFN-τ treatment for 48 h significantly down-regulated the levels of the above five metabolites in the uterus of endometritic mice. Metabolic pathway analysis revealed a total of 3 metabolic pathways identified as significantly associated with endometritis.

Joint analysis of gut flora and metabolomics not only reflects changes in metabolites, but also provides insights into the status of gut flora (Meng et al., 2020). Combined analysis of gut microbiota and metabolomics data suggests that IFN-γ treatment for endometritis may influence metabolic processes by regulating gut microbiota composition and balancing microbial levels. Its mechanism of action may result from the mutual regulation between the gut microbiota and their metabolic products. IFN-τ restored the levels of *Oscillospira* and *Clostridium* flora in the intestine, which in turn regulate the expression of five differential metabolites, and that the metabolic pathways regulated are Arginine biosynthesis, Pyruvate metabolism, Arginine and proline metabolism, and pyruvate metabolism. The metabolic pathways regulated are Arginine biosynthesis, Pyruvate metabolism, Arginine and proline metabolism, which were associated with reduced uterine inflammation.

## Data Availability

The sequencing data of the 16S rRNA gene in this study are available in the Sequence Read Archive (SRA) under project number PRJNA1259142 (https://www.ncbi.nlm.nih.gov/sra/PRJNA1259142). The metabolomics data reported in this paper have been deposited in the OMIX, China National Center for Bioinformation/Beijing Institute of Genomics, Chinese Academy of Sciences (https://ngdc.cncb.ac.cn/omix/release/OMIX010091).
